# Colloidal Organometal Halide Perovskite (MAPbBr_x_I_3−x_, 0≤x≤3) Quantum Dots: Controllable Synthesis and Tunable Photoluminescence

**DOI:** 10.1038/srep35931

**Published:** 2016-10-24

**Authors:** Ying Zhao, Xiangxing Xu, Xiaozeng You

**Affiliations:** 1 State Key Laboratory of Coordination Chemistry, Collaborative Innovation Center of Advanced Microstructures, School of Chemistry and Chemical Engineering, Nanjing University, Nanjing 210093, PR China; 2 School of Chemistry and Materials Science, Nanjing Normal University, Nanjing 210023, PR China

## Abstract

Organic-inorganic perovskite materials, typically methylammonium lead trihalide (MAPbX_3_: MA = methylammonium; X = Br, I), are recently attract enormous attention for their distinguished photo-electronic properties. The control of morphology, composition and dispersability of MAPbX_3_ perovskite nanocrystals is crucial for the property tailoring and still a major challenge. Here we report the synthesis of colloidal MAPbBr_x_I_3−x_(0 ≤ x ≤ 3) nanocrystals at room temperature by using alkyl carboxylate as capping ligands. These nanocrystals exhibit continuously tunable UV-vis absorption and photoluminescence (PL) across the visible spectrum, which is attributed to the quantum confinement effect with certain stoichiometry. Their unique exciton recombination dynamics was investigated and discussed.

Lead halide based perovskites have become famous semiconductor materials for their industrial prospect in solar cells. Up to date, the efficiency of perovskite solar cells has unceasingly boosted up to 22.1%^1^,^2^,^3^,^4^,^5^,^6^,^7^,^8^,^9^. The application potentials of perovskites in light emitting devices^10^,^11^,^12^,^13^,^14^,^15^,^16^,^17^ and lasers^18^,^19^ were also demonstrated. In nanometer scale, the huge specific surface area and thus the abundant interface or surface states of the perovskites exhibit prominent effects on the electronic and photoelectronic properties. Therefore, the challenge emerges for chemists of nano-science and -technology to develop synthesis methods to achieve size-controllable perovskite nanocrystals. In the most recent year, colloidal cesium lead halide perovskites nanocrystals and nanowires were successfully synthesized. Kovalenko *et al*. reported the synthesis of highly luminescent perovskite CsPbX_3_(X = Cl, Br, I) of 4–15 nm, with the photoluminescence tuned within 410−700 nm^20^,^21^. Yang *et al*. developed a solution synthesis of single-crystalline CsPbX_3_(X = Cl, Br, I) nanowires^22^. Prato *et al*. further found that the composition of perovskite nanocrystals can be feasibly tuned by post-synthesis halide anion exchange^23^. Despite the success for the all-inorganic cesium lead halide perovskite colloidal nanocrystals, the synthesis methods of colloidal organometal halide perovskite nanocrystals are comparably less developed. Although there are numerous literatures on preparing organic-inorganic perovskite micro/nanocrystals, most of them are synthesized or grow on substrates. For examples, the synthesis of MAPbI_3_ nanoplates and nanowires on substrates were demonstrated by Jin^24^, Horvath^25^, Grätzel and Park *et al*.^26^. We reported the synthesis of MAPbI_3_ crystals on porous TiO_2_ substrate with the size controllable within 40–700 nm^27^. The substrates served as a scaffold for the precursors were used to control the perovskite growth kinetics^27^,^28^,^29^,^30^,^31^. However, the substrates prevented from scale up the products and the generality for different substrates is limited.

Recently, advances have been made in synthesis of MAPbX_3_(X = Br, I, Cl) nanocrystals in solution without a substrate^32^,^33^,^34^,^35^,^36^. All these reported methods use organic ammonium cation with a long alkyl chain, such as the octylammonium bromide (CH_3_(CH_2_)_7_NH_3_Br) or octadecylammonium bromide (CH_3_(CH_2_)_17_NH_3_Br). The ammonium cation serves as the surface capping ligands of the nanocrystals, limiting the crystal growth in one, two or three dimensions. In this article, we use a different surface modulation strategy by applying alkyl carboxylate, the lead oleate (Pb(CH_3_(CH_2_)_7_CH = CH(CH_2_)_7_COO)_2_, abbreviated as Pb(OA)_2_), as both the lead resource and capping ligands. The as synthesized MAPbBr_x_I_3−x_(0 ≤ x ≤ 3) nanocrystals exhibit continuously tunable UV-vis absorption and photoluminescence (PL) spectra across the visible realm, which is attributed to the size related quantum confinement effect with a fixed stoichiometry of the halide composition.

## Results and Discussion

**Figure 1:**
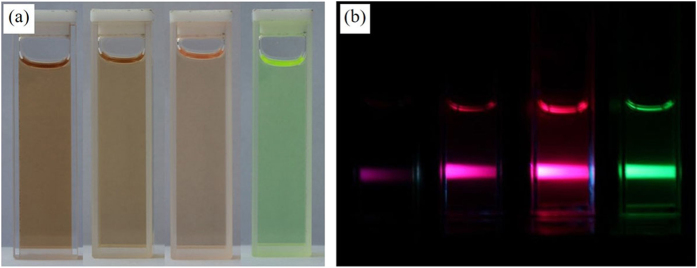
(**a**) Images of typical as synthesized MAPbBr_x_I_3−x_(0 ≤ x ≤ 3) colloidal nanocrystals in solution under the day light and (**b**) images under the ultraviolet excitation (365 nm). From left to right: sample P-I-5, P-I3Br2-5, P-I1Br1-5 and P-Br-5.

The reaction solution contains two solvents, the cyclohexane and the isopropanol. The Pb(OA)_2_ is soluble in the former, and the MAI or MABr (or the mixture) is soluble in the later. When these two solutions are mixed at room temperature, MAPbBr_x_I_3−x_(0 ≤ x ≤ 3) forms immediately which can be easily identified by the color change and the photoluminescence under a portable 365 nm ultraviolet lamp (Fig. 1).

**Figure 2:**
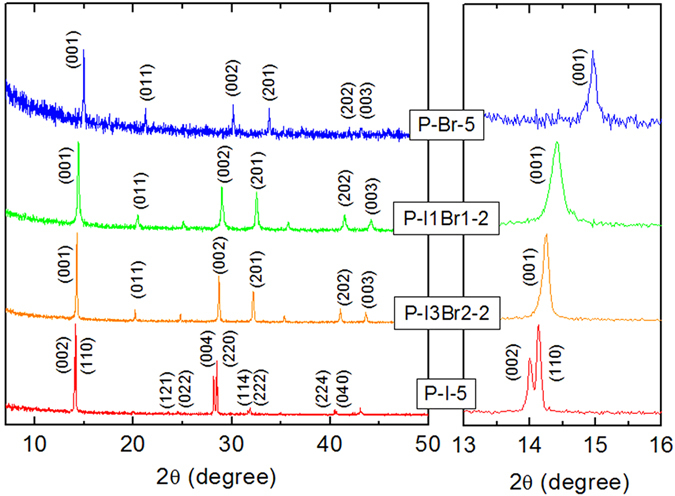
The XRD patterns of some typical as synthesized MAPbBr_x_I_3−x_(0 ≤ x ≤ 3) nanocrystals: sample P-I-5, P-I3Br2-2, P-I1Br1-2 and P-Br-5.

The XRD spectra of the typical products are shown in Fig. 2 (and Figures S1–S4 in Supporting Information). For the samples P-I-2/3/4/5, strong split peaks at 14.0° and 14.1° that respectively corresponding to (002) and (110) crystal plane and the split peaks of the (004) and (220) peaks can be clearly identified. This split feature verified that the as prepared MAPbI_3_ are tetragonal perovskite phase (space group *I*4/*mcm*)^37^,^38^,^39^,^40^,^41^. While for the rest of the samples containing Br^−^, no split can be identified at (001) or (002) peaks, indicating they are cubic phased perovskite (space group *Pm-3m*). The perovskite phases via the regulation of I^−^ and Br^−^ are generally observed by various researchers^38^,^39^,^40^,^41^. For all the samples no impurity of MAI or Pb(OA)_2_ is found. This may due to the fast reaction rate and excess amount of MAI to consume the Pb(OA)_2_. Also none of the other known and related MA_n_PbI_m_ (n, m = 2,4, 3,5, 4,6) phases are observed^41^.

**Figure 3:**
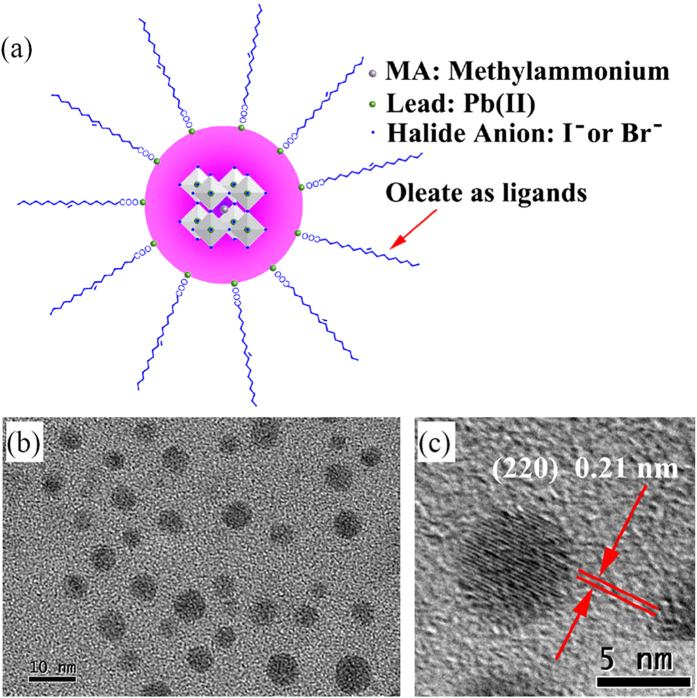
(**a**) Schematic illustration of a perovskite nanocrystals stabilized by oleate as surface ligands, (**b**) A typical TEM image and (**c**) HRTEM image of perovskite nanocrystals of sample P-Br-5.

For all the samples of MAPbBr_x_I_3−x_(0 ≤ x ≤ 3) nanocrystals, the typical FTIR vibration modes of organolead halide perovskites are distinctly presented (Figure S5). The 3300–3000 cm^−1^ broad strong peak is assigned to N-H stretching; the 2950–2820 cm^−1^ peaks are assigned to symmetric and asymmetric stretching vibrations of CH_2_ and CH_3_^36^,^42^,^43^,^44^,^45^; the peak at 1730–1630 cm^−1^ corresponds to the COO^−^ modes and the peak at 1440–1360 cm^−1^ is assigned to the C-H bending^44^. The symmetric O-H stretch and antisymmetric O-H stretch of H_2_O were not found in 3600–3800 cm^−1^ from Figure S5. Thus, no adsorbed H_2_O was detected, suggesting its good temporary stability in ambient air condition. The good dispersability of the perovskites in cyclohexane also suggests that the surface of MAPbBr_x_I_3−x_(0 ≤ x ≤ 3) nanocrystals is coordinated by oleate ligand, like typical oleate or alkylamines modified colloidal nanocrystals. The schematic illustration of a perovskite nanocrystal stabilized by oleate as surface ligands is shown in Fig. 3(a). Figure 3(b,c) are typical TEM and HRTEM images of perovskite nanocrystals of sample P-Br-5, respectively. It indicates that these nanocrystals are well dispersed and the measured crystal lattice matches well with the that of the MAPbBr_3_^32^,^36^. Else TEM images of colloidal perovskite nanocrystals of MAPbBr_x_I_3−x_(0 ≤ x ≤ 3) can be found in Figures S6–S8.

**Figure 4:**
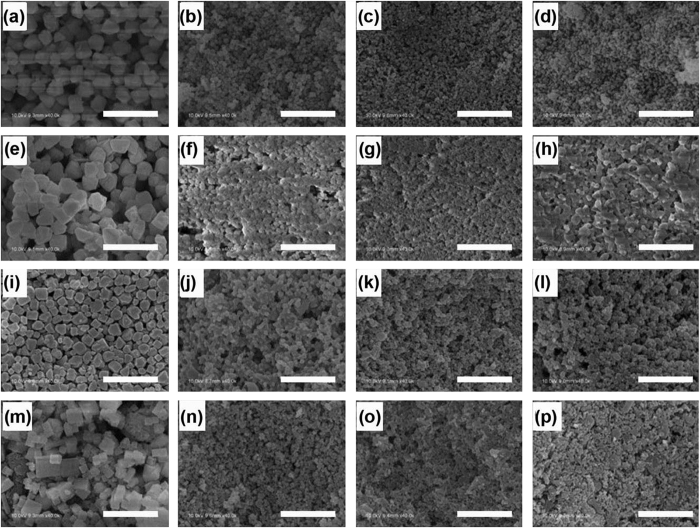
SEM images of four sets of samples (**a**–**d**) P-I-2/3/4/5 from left to right respectively, and the same for (**e**–**h**) P-I3Br2-2/3/4/5, (**i**–**l**) P-I1Br1-2/3/4/5, and (**m**–**p**) P-Br-2/3/4/5 (Scale bar: 1 μm).

The SEM measurement indicates that with the decrement of MA:Pb from 5:1 to 2:1, the size of all the as synthesized MAPbBr_x_I_3−x_(0 ≤ x ≤ 3) nanoparticles increases to ~300 nm (Fig. 4). This trend may find its origin in the nucleation and growth mechanism. Take the nucleation and growth of MAPbI_3_ as an example, the reaction in the solution is as follows:


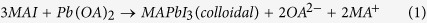


The alkyl carboxylate, lead oleate Pb(OA)_2_, acts as not only as the lead resource to react with MAI, but also as the capping ligands. In addition, the reaction rate is higher with the higher MAI concentration. The faster the nucleation bursts, the more completely Pb(OA)_2_ consumed in solution in a short time, leading to smaller MAPbI_3_ nanocrystals. While in relatively lower MAI concentration, the nucleation rate is slower and the perovskite growth is dominated following the nucleation, resulting bigger MAPbI_3_ nanocrystals. Obviously, it shows significance for all these perovskite materials.

**Figure 5:**
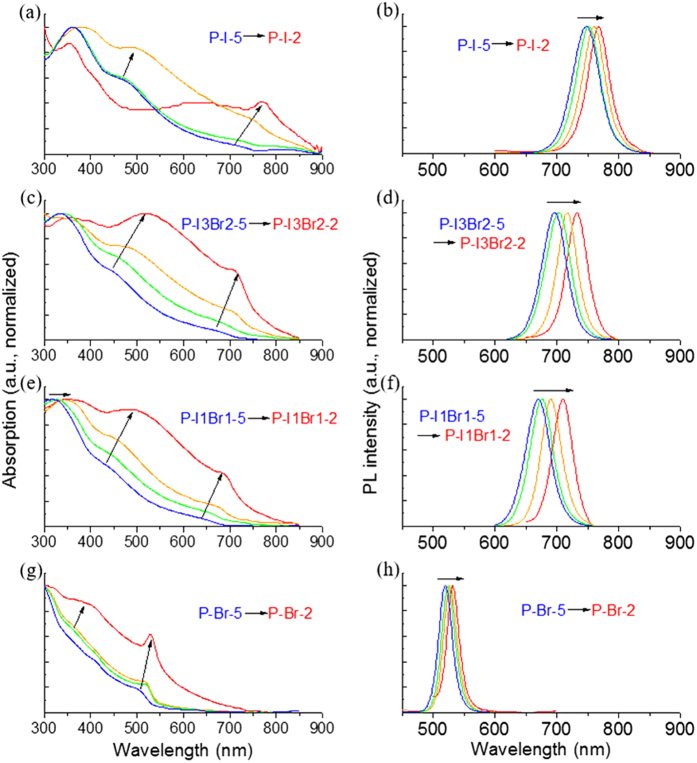
The UV-vis absorption spectra and PL spectra of four sets of samples: (**a**,**b**) P-I-2/3/4/5, (**c**,**d**) P-I3Br2-2/3/4/5, (**e**,**f**) P-I1Br1-2/3/4/5, and (**g**,**h**) P-Br-2/3/4/5. The arrows indicate the spectrum evolution of the samples (Excitation: 365 nm).

**Figure 6:**
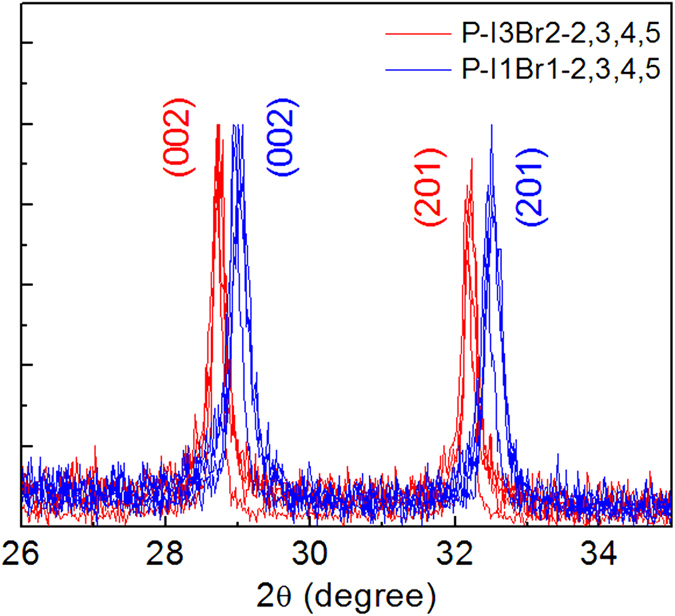
The XRD peaks of (002) and (201) for P-I3Br2-2/3/4/5 and P-I1Br1-2/3/4/5.

The UV-vis absorption spectra and corresponding PL spectra of four sets of samples (P-I-2/3/4/5, P-I3Br2–2/3/4/5, P-I1Br1-2/3/4/5, P-Br-2/3/4/5) are shown in Fig. 5. The band edge absorption peak locates at 764 nm for big MAPbI_3_ nanocrystals (P-I-2, ~300 nm by SEM). It undergoes a blue shift to 735 nm for smaller MAPbI_3_ nanocrystals (P-I-5, ~5 nm by TEM). Accordingly, the photoluminescence (PL) peak shifts from 767 nm to 747 nm with the size decreasing. The synthesized MAPbBr_3_ and MAPbBr_x_I_3−x_(0 ≤ x ≤ 3) nanocrystals also show similar UV-vis absorption and PL spectra dependent on the size. This size related monotonic blue shift of both spectra can be induced by the intrinsic quantum confinement effect, for the small crystal size is comparable to the Bohr diameter of MAPbBr_x_I_3−x_(0 ≤ x ≤ 3)^46^,^47^,^48^,^49^,^50^. There remains only one concern, that since the spectra of P-I3Br2-2/3/4/5 and P-I1Br1-2/3/4/5 are over lapped, it is doubtful if the spectrum shift is caused by the component deviation. By comparing the XRD peaks of P-I3Br2-2/3/4/5 and P-I1Br1-2/3/4/5, this possibility is excluded. As shown in Fig. 6, the (002) and (201) peaks of each set are separated from each other, while almost fixed for a same set. It suggests that the perovskites of the same sets have the same crystal parameter, thus have the same component. Also, the widening of the XRD peaks indicates the size of nanocrystals decreases (Figures S1–S3). These features further support the intrinsic quantum confinement effect for the observed spectra shift.

The PL lifetimes of MAPbBr_x_I_3−x_(0 ≤ x ≤ 3) were measured (Figures S9–11) to get insight into the exciton recombination dynamics. These PL decays can be well fitted by the bi-exponential function (Figure S12):


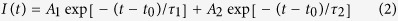


where *τ*_1_ and *τ*_2_ are the fitted decay lifetimes; *A*_1_ and *A*_2_ are the weighting parameters. The average lifetime *τ*_ave_ is calculated by equation (3):


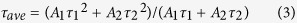


The results are shown in Table 1. It suggests two dynamics, a fast decay (*τ*_1_) and a longer-lived component (*τ*_2_). With the decreasing size of the nanocrystal or the blue shift of the PL peak, the *τ*_ave_ increases for samples of P-I-2/3/4/5, P-I1Br1-2/3/4/5 and P-Br-2/3/4/5. For sample P-I-2, it has a very short lifetime *τ*_ave_ of 3.0 ns. The lifetime of the sample P-I-5 is incredibly prolonged to 166.2 ns. This lifetime range covers the ever reported lifetimes of tetragonal MAPbI_3_ in the form of nanowire, nanorod, film or bulk (Table 2). A very short lifetime of P-I-2 indicates that there exist high effective nonradiative recombination channels in the material^50^,^51^,^52^. It is proposed to be chemical or structural defects in the MAPbI_3_ nanocrystals. The long lifetime of the sample P-I-5 is unexpected, because it has a big specific surface area which may induce vast surface states. Possibly it suggests that the surface of MAPbI_3_ nanocrystals is well passivated by the oleate ligands^53^,^54^,^55^. It was noticed that from P-I-2 to P-I-5, both *τ*_1_ and *τ*_2_ increase drastically. The fast decay *τ*_1_ of P-I-5 is 25.5 ns, a value much bigger than the slow decay *τ*_2_ of P-I-2 4.8 ns. The mechanisms how the *τ*_1_ and *τ*_2_ are separately or cohesively tuned are presently unclear. However, it is not likely to originate from quantum confinement effect, but more possible related to the surface or defect states and charge carrier delocalization in MAPbI_3_^20^. For the samples from P-I1Br1-2 to P-I1Br1-5, the lifetime *τ*_ave_ boosts from 1.1 to 42.1 ns. While for the samples from P-Br-2 to P-Br-5, the lifetime *τ*_ave_ only increases from 13.0 to 17.1 ns, with the small PL peak shift from 532 to 525 nm. Compared with MAPbBr_3_ single crystal or film (Table 2), these as prepared MAPbBr_3_ nanocrystals exhibit shorter lifetime, suggesting more nonradiative recombination effect involved^13^,^56^,^57^. Unfortunately, there was no lifetime data of MAPbBr_x_I_3−x_(0 ≤ x ≤ 3) quantum dots synthesized by using ammonium cation with a long alkyl chain as surface capping ligands^35^,^36^,^37^,^38^,^39^. Further investigation on how surface ligands type would affect the fluorescence lifetime may give us a deeper understanding of the exciton recombination dynamics, and thus better control of the PL for future use in construction of devices.

## Conclusions

Organometal halide perovskite MAPbBr_x_I_3−x_(0 ≤ x ≤ 3) colloidal nanocrystals were synthesized by mixing Pb(OA)_2_ cyclohexane solution and MAX(X = I or/and Br) isopropanol solution. The size of the perovskites was successfully controlled by the oleate as the ligands, and also by the Pb:MA ratio. The UV-vis absorption and PL spectra show a blue shift as the size decreasing monotonically, which is ascribed for quantum confinement effect. Significantly, these colloidal organometal halide perovskite nanocrystals were dispersed well in nonpolar organic solvent, e.g. toluene or cyclohexane. This would be useful for fabricating perovskite thin films in various substrates such as silicon, polymer or glass. In addition to the method could be generally applied to synthesize other organometal halide perovskite materials, this study would bring chances to the design and fabrication of new photovoltaic and electronic devices.

## Materials and Methods

### Materials

Lead acetate trihydrate (AR), sodium oleate (>99.5%) and cyclohexane (>99.5%) were bought from Sinopharm. Isopropanol (anhydrous, 99.5%) was purchased from J&K. Hydroiodic acid (HI, 55–58%), hydrobromic acid (HBr, 47.0%) were purchased from Sigma-Aldrich. Methylamine (40% in methanol) was bought from TCI. All chemicals were used as received unless specified otherwise.

### Synthesis of Pb(OA)_2_ (OA = Oleic acid)

The lead acetate solution (1.037 g in 5 mL H_2_O) was added into the solution of sodium oleate (1.90 g in 25 mL mixture of ethanol and H_2_O v:v = 1:1) with vigorous stirring. The precipitate (Pb(OA)_2_) was separated by centrifugation, washed with water and finally dried in oven at 85 °C overnight.

### Synthesis of MAX (MA = methylammonium, X = I, Br)

Hydroiodic acid (10 mL, 0.075 mol) or hydrobromic acid (8.6 mL, 0.075 mol) was added to a solution of excess methylamine (24 mL, 0.192 mol) dropwisely at 0 °C under stirring. The mixture was continuously stirred in the ice bath for 2 hrs. The raw product (MAI or MABr) was obtained after evaporation and dried under dynamic vacuum at 60 °C for 12 hrs. The purified MAI or MABr was obtained by recrystallization from a mixed solvent of diethyl ether and ethanol.

### Synthesis of MAPbBr_x_I_3−x_(0 ≤ x ≤ 3) nanocrystals

Typically, Pb(OA)_2_ (15.4 mg, 0.02 mmol) was dissolved in cyclohexane (20 mL) to form a solution A. MAI (6.4 mg, 0.04 mmol) was dissolved in isopropanol (20 mL) to make a solution B. Solution A was injected into to solution B with volume ratio of 1:1. The perovskite MAPbI_3_ nanocrystals can be isolated by centrifugation (3 min at 14000 rpm) and redispersed in solvent of cyclohexane or toluene. The MAPbBr_x_I_3−x_(0 ≤ x ≤ 3) nanocrystals was synthesized similarly, by introducing MABr to the reaction. The samples are numbered in the form of P-I-*y*, P-I*a*Br*b*-*y*, or P-Br-*y*, where P = perovskite; *a* and *b* are the I:Br ratio of *a*:*b* such as I1Br1 or I2Br3; *y* = 2, 3, 4 or 5 means the MA:Pb ratio of y:1 (Table S1). “P-I-2/3/4/5” means four samples of different *y* values: P-I-2, P-I-3, P-I-4 and P-I-5. To eliminate the effects from the solvent, the volume ratio of cyclohexane to isopropanol is controlled as a constant of 1:1 for all the reactions in the context. The Pb(OA)_2_ is kept as a constant. The concentration of MA in isopropanol is investigated as a variable.

### Instruments

The powder X-ray Diffraction (XRD) spectra were performed on a Bruker D8 Advance instrument with a Cu Kα radiation (λ = 1.5418 Å). The scanning electron microscopy (SEM) images were obtained on the S-4800 SEM at 20 kV. The transmission electron microscopy (TEM) images were measured on a HT7700 (Hitachi) TEM at an acceleration voltage of 100 kV. The FTIR spectra were recorded on a Vector 22 spectrometer with a resolution of 2 cm^−1^ by using KBr pellets. The absorption spectra were obtained by a Shimadzu UV-2700 UV/Vis spectrophotometer. The PL spectra were collected with a Hitachi F-4600 fluorescence spectrophotometer. PL lifetimes were measured with a Zolix Omini-λ 300 fluorescence spectrophotometer and a picosecond pulsed diode laser (Edinburgh Instruments Ltd.) operating at 379.2 nm.

## Additional Information

**How to cite this article**: Zhao, Y. *et al*. Colloidal Organometal Halide Perovskite (MAPbBr_x_I_3−x_, 0≤x≤3) Quantum Dots: Controllable Synthesis and Tunable Photoluminescence. *Sci. Rep.* **6**, 35931; doi: 10.1038/srep35931 (2016).

## Supplementary Material

Supplementary Information

## Figures and Tables

**Table 1:** The fitting decay lifetimes of τ_1_ and τ_2_, the average lifetime τ_ave_ and corresponding PL peak.

Sample	τ_1_ (ns)	τ_2_ (ns)	τ_av_ (ns)	PL λ_Peak_ (nm)
**P-I-2**	1.2	4.8	3.0	768
**P-I-3**	2.7	34.3	18.3	760
**P-I-4**	3.5	87.3	66.8	752
**P-I-5**	25.5	201. 7	166.2	748
**P-I1Br1-2**	0.47	4.1	1.1	710
**P-I1Br1-3**	0.46	15.1	10.6	690
**P-I1Br1-4**	5.0	47.0	35.9	676
**P-I1Br1-5**	6.8	55.6	42.1	670
**P-Br-2**	0.85	16.8	13.0	532
**P-Br-3**	2.9	24.5	16.9	527
**P-Br-4**	2.8	25.2	17.0	526
**P-Br-5**	3.0	24.1	17.1	525

**Table 2:** Reported decay lifetimes and corresponding PL peaks for MAPbI_3_ and MAPbBr_3_, T = tetragonal, C = cubic.

Material	Size/shape/form	Lifetime (ns)	PL λ_Peak_ (nm)	Phase
MAPbI_3_	bulk^26^	30	775	T
	nanowires (*d* = 100 nm)^26^	45	765	T
	film (800 nm)^58^	80	774	T
	bulk 0.3 × 2 μm^34^	9	762	T
	wires 1500 × 34 nm^34^	30	756	T
	rods 810 × 54 nm^34^	66	760	T
	film^59^	9.6	~770	—
	films (20 μm^2^)^60^	~5	~751	—
MAPbBr_3_	single crystal^61^	41, 57	570	C
	film^61^	13, 168	560	C
	film^62^	100	530	C
	nanocrystal 3.3 nm^36^	13.5	515	C
