# Supplementary material for: Colloidal Organometal Halide Perovskite (MAPbBrxI3−x, 0≤x≤3) Quantum Dots: Controllable Synthesis and Tunable Photoluminescence
Source: Sci Rep. 2016 Oct 24;6:35931. doi: 10.1038/srep35931 (PMC5075927; doi:10.1038/srep35931)
Supplement: Supplementary Information [file srep35931-s1.doc]

Supplementary Information

**Colloidal Organometal Halide Perovskite (MAPbBr_x_I_3-x_, 0≤x≤3) Quantum Dots: Controllable Synthesis and Tunable Photoluminescence**

Ying Zhao,^1^ Xiangxing Xu^2^,*& Xiaozeng You^1^,*

^1^ State Key Laboratory of Coordination Chemistry, Collaborative Innovation Center of Advanced Microstructures, School of Chemistry and Chemical Engineering, Nanjing University, Nanjing 210093, PR China

^2^ School of Chemistry and Materials Science, Nanjing Normal University, Nanjing 210023, PR China.

* Corresponding Author: xuxx@njnu.edu.cn; youxz@nju.edu.cn

**Table S1.** The synthesis control of the MAPbBr_x_I_3-x_(0≤x≤3) nanocrystals.

| **Samples** | **MAI (mmol)** | **MABr (mmol)** |  | **Pb(OA)_2_ (mmol)** |  | **Pb(OA)_2_: MA** |
| --- | --- | --- | --- | --- | --- | --- |
|  | in 20 ml isopropanol | |  | in 20 ml cyclohexane |  | (molar ratio) |
| **P-I-2** | 0.04 | - |  | 0.02 |  | 1:2 |
| **P-I-3** | 0.06 | - |  | 0.02 |  | 1:3 |
| **P-I-4** | 0.08 | - |  | 0.02 |  | 1:4 |
| **P-I-5** | 0.10 | - |  | 0.02 |  | 1:5 |
| **P-Br-2** | - | 0.04 |  | 0.02 |  | 1:2 |
| **P-Br-3** | - | 0.06 |  | 0.02 |  | 1:3 |
| **P-Br-4** | - | 0.08 |  | 0.02 |  | 1:4 |
| **P-Br-5** | - | 0.10 |  | 0.02 |  | 1:5 |
| **P-I1Br1-2** | 0.02 | 0.02 |  | 0.02 |  | 1:2 |
| **P-I1Br1-3** | 0.03 | 0.03 |  | 0.02 |  | 1:3 |
| **P-I1Br1-4** | 0.04 | 0.04 |  | 0.02 |  | 1:4 |
| **P-I1Br1-5** | 0.05 | 0.05 |  | 0.02 |  | 1:5 |
| **P-I3Br2-2** | 0.024 | 0.016 |  | 0.02 |  | 1:2 |
| **P-I3Br2-3** | 0.036 | 0.024 |  | 0.02 |  | 1:3 |
| **P-I3Br2-4** | 0.048 | 0.032 |  | 0.02 |  | 1:4 |
| **P-I3Br2-5** | 0.06 | 0.04 |  | 0.02 |  | 1:5 |

**Figure S1.** The XRD patterns of the samples P-I3Br2-2/3/5.

**Figure S2.** The XRD patterns of the samples P-I1Br1-2/3/5.

**Figure S3.** The XRD patterns of the samples P-Br-2/3/5.

**Figure S4.** The XRD patterns of the samples P-I-2/3/5.

**Figure S5.** FTIR spectrum of the samples P-I-2, P-I1Br1-2 and P-Br-2.


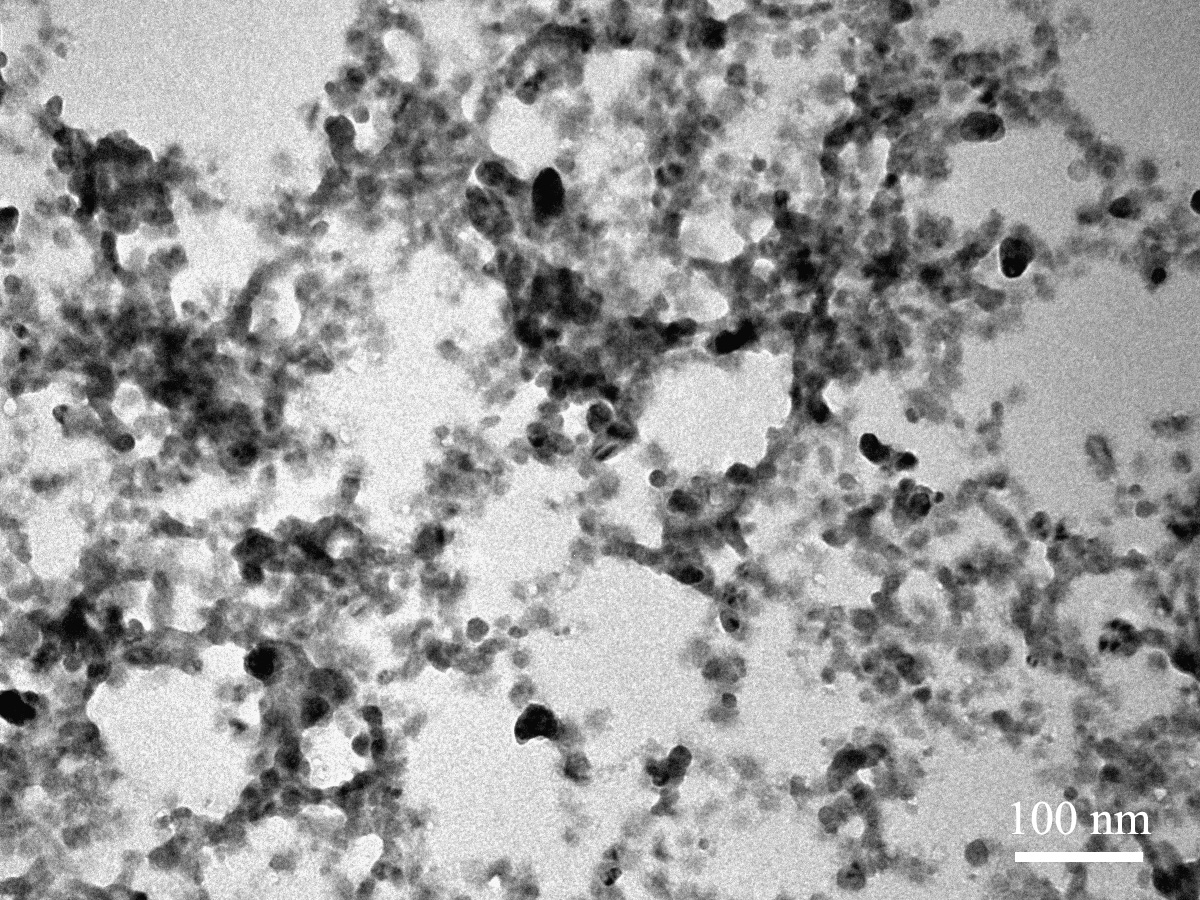


**Figure S6.** A typical TEM image of the sample P-I-5.


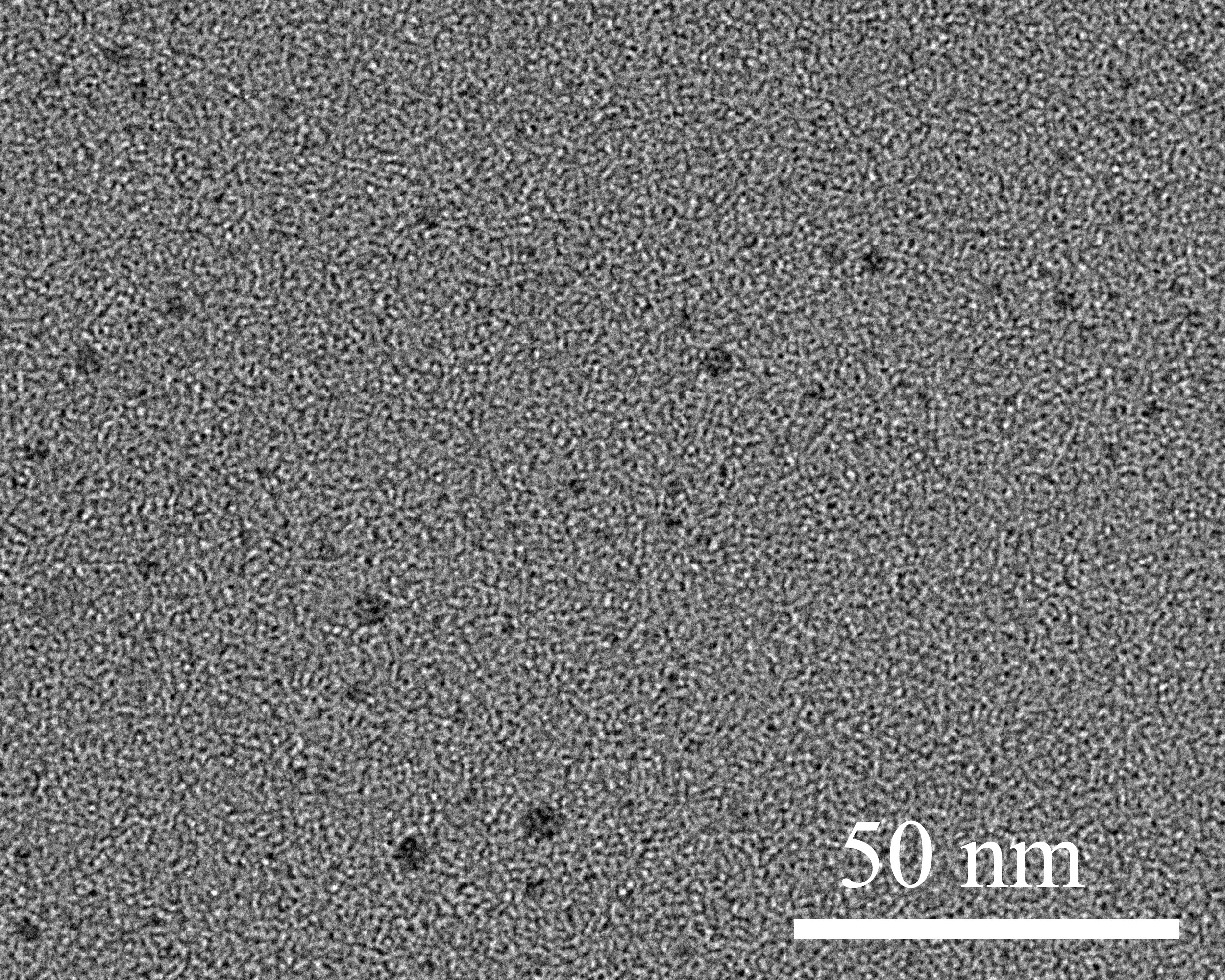


**Figure S7.** A typical TEM images of the sample P-I1Br1-5.


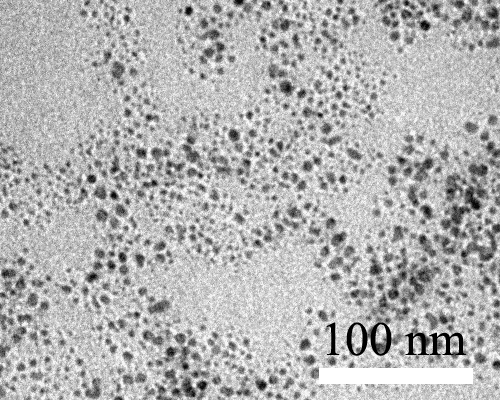


**Figure S8.** A typical TEM images of the sample P-I3Br2-5.

**Figure S9.** Time-resolved PL decay detected at the peak wavelength of emission for the samples P-I-2/3/4/5.

**Figure S10.** Time-resolved PL decay detected at the peak wavelength of emission for the samples P-I1Br1-2/3/4/5.

**Figure S11.** Time-resolved PL decay detected at the peak wavelength of emission for the samples P-Br-2/3/4/5.

**Figure S12.** Time-resolved PL decay of the samples P-I-5, together with the fitting curves. The bi-exponential fit is obviously better than the exponential fit profile. While the tri-exponential fit does not perform better than the bi-exponential fit.
